# Epigenetic programming reshapes innate immune memory: decoding the molecular imprint of gouty inflammation

**DOI:** 10.3389/fphar.2025.1678958

**Published:** 2025-11-12

**Authors:** Wenjie Su, Yifan Lu, Zhiqiang Luo, Hui Xiong

**Affiliations:** 1 Hunan University of Chinese Medicine, Changsha, Hunan, China; 2 Department of Orthopedics, The First Affiliated Hospital of Hunan University of Chinese Medicine, Changsha, Hunan, China; 3 Department of Orthopedics, The Second Affiliated Hospital of Hunan University of Chinese Medicine, Changsha, Hunan, China; 4 Department of Orthopedics, Xiangtan Hospital of Traditional Chinese Medicine, Xiangtan, Hunan, China

**Keywords:** gout, epigenetic reprogramming, trained immunity, DNA methylation, histonemodification, acetylation, non-coding RNA

## Abstract

Gout is an inflammatory joint disease caused by abnormal uric acid metabolism, characterized by the deposition of urate crystals in joints and surrounding tissues, leading to acute or chronic inflammatory responses. The etiology and pathogenesis of gout are complex, and there is currently a lack of ideal therapeutic drugs and treatment strategies. Epigenetic modifications influence and regulate gene function and characteristics through mechanisms such as DNA methylation, histone modifications, chromatin remodeling, and non-coding RNA, thereby exerting significant effects on the physiological and pathological states of the body. Recently, epigenetic modifications and trained immunity in gout have garnered increasing research interest. Epigenetic modification-mediated trained immunity represents a frontier area in the study of gout pathogenesis. This review summarizes the latest findings on the role and regulatory mechanisms of epigenetic modifications in the development of gout, as well as the role of epigenetic remodeling-mediated trained immunity in gout and the potential applications of epigenetic intervention strategies in gout, providing new insights into the relationship between persistent inflammation, epigenetics, and innate immune memory in gout.

## 1 Introduction

Gout is a common crystal-induced joint disease with a clinical history dating back to ancient times, often referred to as the “disease of kings”. Elevated uric acid levels are associated with increased purine intake (Straton et al., 2024),characterized by the deposition of monosodium urate (MSU) crystals within or around joints, tendons, bursae, and other tissues, leading to painful recurrent attacks and tissue damage, thereby impairing the quality of life for patients with this condition (FitzGerald, 2025; Yuan et al., 2025). The incidence and prevalence of gout are closely associated with the prevalence of obesity and metabolic syndrome worldwide (Scuiller et al., 2020). The prevalence of gout is increasing globally, being more common in developed countries than in developing countries. Additionally, gout is more prevalent in men than in women, and hyperuricemia plays the most significant role in the development of gout (Asghari et al., 2024). However, not all patients with elevated serum uric acid levels develop the disease (Grassi et al., 2013). Hyperuricemia is a necessary condition for the occurrence of gout, but not a sufficient condition, only10%–15% of patients with hyperuricemia develop clinical manifestations of the disease. Gout appears to be a multifactorial metabolic disorder, and its pathogenesis should not be attributed solely to hyperuricemia or monosodium urate (MSU) crystals (Bardin and Richette, 2014; Zhang, 2021). Without treatment, gout attacks typically resolve spontaneously within 7–14 days. Following resolution, there is a pain-free, asymptomatic period (intercritical gout) until the next gout attack occurs. Over time, some patients with persistent hyperuricemia may develop tophus, chronic gouty arthritis (persistent joint inflammation induced by monosodium urate crystals), and structural joint damage (Dalbeth et al., 2021). Current treatment for gout includes anti-inflammatory therapy and long-term measures to lower uric acid levels (Forster, 2024). Standard pharmacological treatment for gout attacks includes colchicine, NSAIDs, and the option of oral or intramuscular corticosteroids, with IL-1 inhibitors newly identified as an option for refractory attacks resistant to standard therapy (Yuan et al., 2025). However, traditional drug formulations cannot target inflammatory lesions and may rapidly inactivate in the systemic circulation, leading to reduced efficacy and systemic side effects (Peng et al., 2023). Therefore, identifying new mechanisms and targets for treating gout is necessary.

As research has progressed, it has been discovered that hyperuricemia and gout are complex diseases mediated by the interaction of genetic, epigenetic, and environmental exposures (Zhao et al., 2022). Studies have shown that over 80% of disease-associated variants are located in the non-coding regions of the genome (Cai et al., 2022). Therefore, it has been proposed that changes in gene expression, rather than changes in the genetic code sequence, are more likely to influence the development of gout. With the advancement of technology, significant progress has been made in the exploration of epigenetics. Epigenetics regulates gene expression and has been shown to play a key role in various metabolic diseases, such as gout, diabetes, obesity, non-alcoholic fatty liver disease (NAFLD), osteoporosis, hyperthyroidism, and hypothyroidism (Wu et al., 2023). The epigenome is an important mechanism for regulating gene expression. Notably, trained immunity is the result of epigenomic remodeling, which can make innate immune cells more sensitive to MSU crystals, directly related to gout (Leask et al., 2024). In gout, innate immune cells play a central role in initiating and maintaining inflammatory responses, followed by proteomic and epigenetic modifications (Straton et al., 2024). Over time, a significant body of literature has emerged elucidating how the immune system mediates inflammatory responses to urate and urate crystals (Saeed et al., 2014; Joosten et al., 2020). Over the past decade, increasingly compelling evidence has demonstrated that innate immune cells can acquire adaptive characteristics, leading to long-term functional changes. This *de facto* innate immune memory is referred to as trained immunity, and the regulation of trained immunity may offer new approaches for treating inflammatory diseases (Netea and Joosten, 2024). Trained immunity is a functional state of the innate immune response characterized by long-term epigenetic reprogramming of innate immune cells. While trained immunity is beneficial against infection, inappropriate induction by endogenous stimuli can lead to abnormal inflammation (Ochando et al., 2023). Recent studies have demonstrated that soluble urate can induce long-term epigenetic reprogramming in bone marrow cells to induce “trained immunity,” while the aftermath of urate is believed to mediate most of the physiological effects of hyperuricemia and gout (Keenan, 2020). Therefore, epigenetic reprogramming-mediated trained immunity epigenetically reprogrammed trained immunity is a critical component in disease treatment.

Currently, no review articles exist on epigenetic remodeling mediating trained immunity in gout. This knowledge gap presents two major research challenges: First, the theoretical framework for the chronicity mechanism of gout remains incomplete, failing to explain individual variations. Second, clinical treatment strategies remain limited to anti-inflammatory or urate-lowering approaches, lacking interventions targeting immune memory. Given this, this review aims to elucidate the latest discoveries regarding the role and regulatory mechanisms of epigenetic modifications in gout progression. It also summarizes the role of epigenetically mediated trained immunity in gout and the potential applications of epigenetic intervention strategies in gout, offering new perspectives for understanding persistent inflammation, epigenetics, and innate immune memory in gout. This work not only fills a theoretical gap in gout immune memory research but also integrates epigenetic regulation with trained immunity theory, opening new avenues to overcome current therapeutic bottlenecks. It will provide novel targets for developing precision therapies targeting epigenetic editing, advancing gout treatment from symptom management toward etiological intervention.

## 2 Epigenetic mechanisms in gout

Gout is a form of arthritis caused by an inflammatory response resulting from the deposition of monosodium urate (MSU) crystals in the synovial fluid of joint spaces. It is characterized by acute inflammation in affected joints (known as a gout flare), with asymptomatic intervals in between (Wolyncewicz et al., 2021). However, only 10%–15% of individuals with hyperuricemia develop gout, indicating the presence of additional risk factors. Several studies have provided updated information on the influence of environmental and genetic factors on the progression of clinical gout. Elevated urate concentrations and exposure to various external factors can trigger gout attacks, highlighting the potential relationship between epigenetic mechanisms and gout inflammation (Straton et al., 2024). Recently, genetic and epigenetic genome-wide studies have revealed new pathways in the inflammatory process of gout, including genetic associations with epigenetic modification factors. Epigenome-wide association studies have also shown epigenetic remodeling in gout, which may regulate the innate immune system’s responsiveness to uric acid monosodium crystals (Leask et al., 2024).

The term “epigenetics” was first coined in 1942, and with advancements in technology, research in epigenetics has made significant progress. There are four primary epigenetic mechanisms, including DNA methylation, histone modifications, chromatin remodeling, and non-coding RNA (ncRNA) (Wu et al., 2023). Epigenetics is a fundamental gene-environment interaction process. Unlike genomic modifications, epigenetic modifications are more universal and reversible (Cai et al., 2022). Studies have shown that urate-induced epigenetic changes in myeloid cells may serve as therapeutic targets for gout (Badii et al., 2021). An interesting finding was the identification of XDH loci associated with urate levels, particularly in males, attributed to its expression in urinary epithelial cells. Furthermore, this study provides new insights into epigenetic plasticity as a potential factor in gout by revealing gout-associated loci involved in epigenetic control of gene expression (Major et al., 2024). Thus, elucidating the role of epigenetics in gout has become a current priority.

### 2.1 Role of DNA methylation in gout

DNA methylation is the most common post-replication DNA modification in mammals and is therefore one of the most extensively studied epigenetic modifications (Aristizabal et al., 2020). It is now widely recognized that DNA methylation, in conjunction with other regulatory factors, is a major epigenetic factor influencing gene activity (Moore et al., 2013). DNA methylation refers to the transfer of a methyl group to the C5 position of cytosine by DNA methyltransferases, resulting in the formation of 5-methylcytosine. DNA methylation regulates gene expression by recruiting proteins involved in gene suppression or inhibiting the binding of transcription factors to DNA (Moore et al., 2013). Typically, DNA methylation occurs in CpG islands within the promoter regions of housekeeping genes, where guanine dinucleotides (CpG) are highly concentrated (Karouzakis et al., 2018; Lim et al., 2019). DNA methylation is a common epigenetic mechanism used by cells to regulate gene expression. Low-methylated promoter DNA is associated with active transcription, while high-methylated promoter DNA leads to reduced transcription (Tseng et al., 2020). Methyltransferases that influence DNA methylation are primarily classified into three categories: DNMT1, DNMT3a, and DNMT3b. Additionally, DNMT1 is the primary enzyme responsible for maintaining methylation patterns during DNA replication, while DNMT3A and DNMT3B primarily function as *de novo* methyltransferases, generating new methylation patterns (Zhong et al., 2016).

Xiaowu Zhong et al. screened 336 gout patients and 306 healthy control subjects (from the South China population) for DNMT1, DNMT3A, and DNMT3B polymorphisms in 336 gout patients and 306 healthy control subjects (from the South China population). The results indicated that the DNMT1 rs2228611 polymorphism may be involved in the pathogenesis of gout, leading to changes in DNMT1 enzyme activity and potentially affecting DNA methylation status (Zhong et al., 2016). SLC2A9 and ABCG2 are major genetic loci associated with uric acid and gout across multiple ancestral populations. A cross-ancestry meta-analysis of GWAS cohorts of European (EUR) or East Asian (EAS) descent identified single-nucleotide polymorphisms (SNPs) in SLC2A9 (uric acid: rs3775948; gout: rs4697701) and ABCG2 (gout: rs2622621) at single-variant resolution. Cross-ancestry fine-mapping has identified ancestral and functional variants in SLC2A9 or ABCG2 that exhibit primate-specific regulatory effects on uric acid and gout (Takei et al., 2021). A cross-racial meta-analysis of serum uric acid epigenome-wide association studies (EWAS) revealed that SLC2A9 is the most influential serum uric acid locus, with five CpGs associated with SLC2A9 gene expression. Four CpG sites in SLC2A9 have a significant causal effect on serum uric acid levels and/or gout. Additionally, the significant causal effect of CpG cg03725404 in SLC2A9 has been proven to be associated with gout (Tin et al., 2021). Through comprehensive analysis of genome-wide genotype and DNA methylation data, nuclear receptor-binding protein 1 (NRBP1) was identified as a gout risk gene, with its regulatory region (72 bp upstream of the transcription start site, referred to as B1). DNA methylation increases the binding of transcription factor TFAP2A to B1, leading to suppressed gene expression; low methylation in the NRBP1 promoter region reduces TFAP2A binding, thereby increasing NRBP1 expression, which may contribute to the development of gout (Zhu et al., 2017). Another study showed that overexpression of NRBP1 significantly reduced the mRNA and protein expression of GLUT9 and URAT1, while knockdown of NRBP1 significantly increased the mRNA and protein expression of ABCG2. NRBP1 inhibition plays an important role in alleviating hyperuricemia and gout by upregulating ABCG2 through the Wnt/β-catenin signaling pathway in HK-2 cells (Zhang Q. et al., 2023). Additional studies have shown that gout is associated with UMOD gene mutations (Gibson, 2012). UMOD gene variants are associated with susceptibility to chronic kidney disease risk (Köttgen et al., 2009). UMOD hypermethylation is significantly associated with gout risk in Chinese male patients. Furthermore, UMOD methylation levels can serve as predictive biomarkers for gout risk. Urinary regulin glycoprotein (UMOD) is a methylation-dependent protein, and due to its high methylation status, its expression is higher in gout patients than in control groups (Yang et al., 2017).

Another interesting study links the dopaminergic system to renal function, with dopamine regulating renal function (Zeng et al., 2007), inducing glomerular filtration (Sulikowska et al., 2008), subsequently reducing uric acid levels and excretion rates. Dopamine degradation may increase the reabsorption of sodium and uric acid salts, ultimately leading to gout (Ying et al., 2019). The human COMT gene encodes catechol-O-methyltransferase (COMT), which is highly expressed in proximal tubular epithelial cells, the Henle’s loop of Henle, and the ureter (Azhar et al., 2020). COMT is involved in the metabolism of estradiol and catecholamines (such as epinephrine, norepinephrine, and dopamine) (Hassan et al., 2022). Research indicates that the COMT rs4680 (Val158Met) variant may prevent gout and potentially influence gout through regulation of dopamine levels (Dong et al., 2015). There is a significant association between COMT hypomethylation and the risk of gout in men, and COMT hypomethylation may serve as a potential diagnostic biomarker for gout in the future (Ying et al., 2019).

CC chemokine ligand 2 (CCL2) is a chemokine involved in the recruitment and migration of monocytes/macrophages. Previous studies have demonstrated that the CCL2 −2518A/G (rs1024611) single nucleotide polymorphism (SNP) plays a key role in the susceptibility to gout in Chinese Han male populations (Sun R. et al., 2015). Studies on the contribution of chemokine CCL2 in the progression of gout have revealed a positive correlation between low methylation in the promoter region and enhanced inflammatory responses in gout among Chinese Han men (Li et al., 2017). In summary, methylation of gout-related genes is closely associated with the pathogenesis and progression of gout.

**FIGURE 1:**
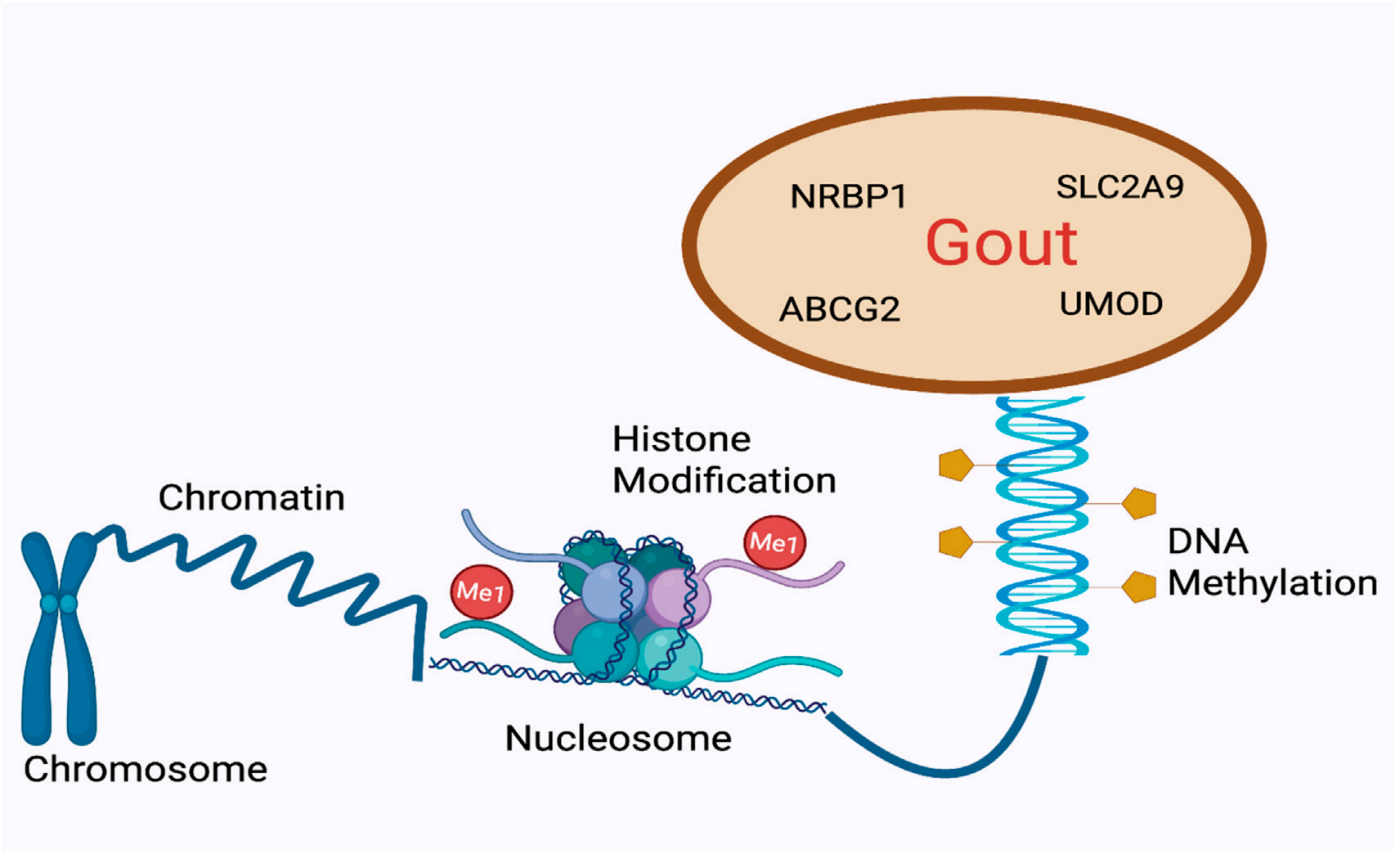
DNA methylation and gout. Changes in the methylation status of gout-related are involved in regulating the pathogenesis and progression of gout.

In conclusion, these findings suggest that alterations in gout factor methylation are associated with the pathogenesis and progression of gout (Figure 1).

### 2.2 Histone modifications and chromatin remodeling in gout inflammation

**FIGURE 2:**
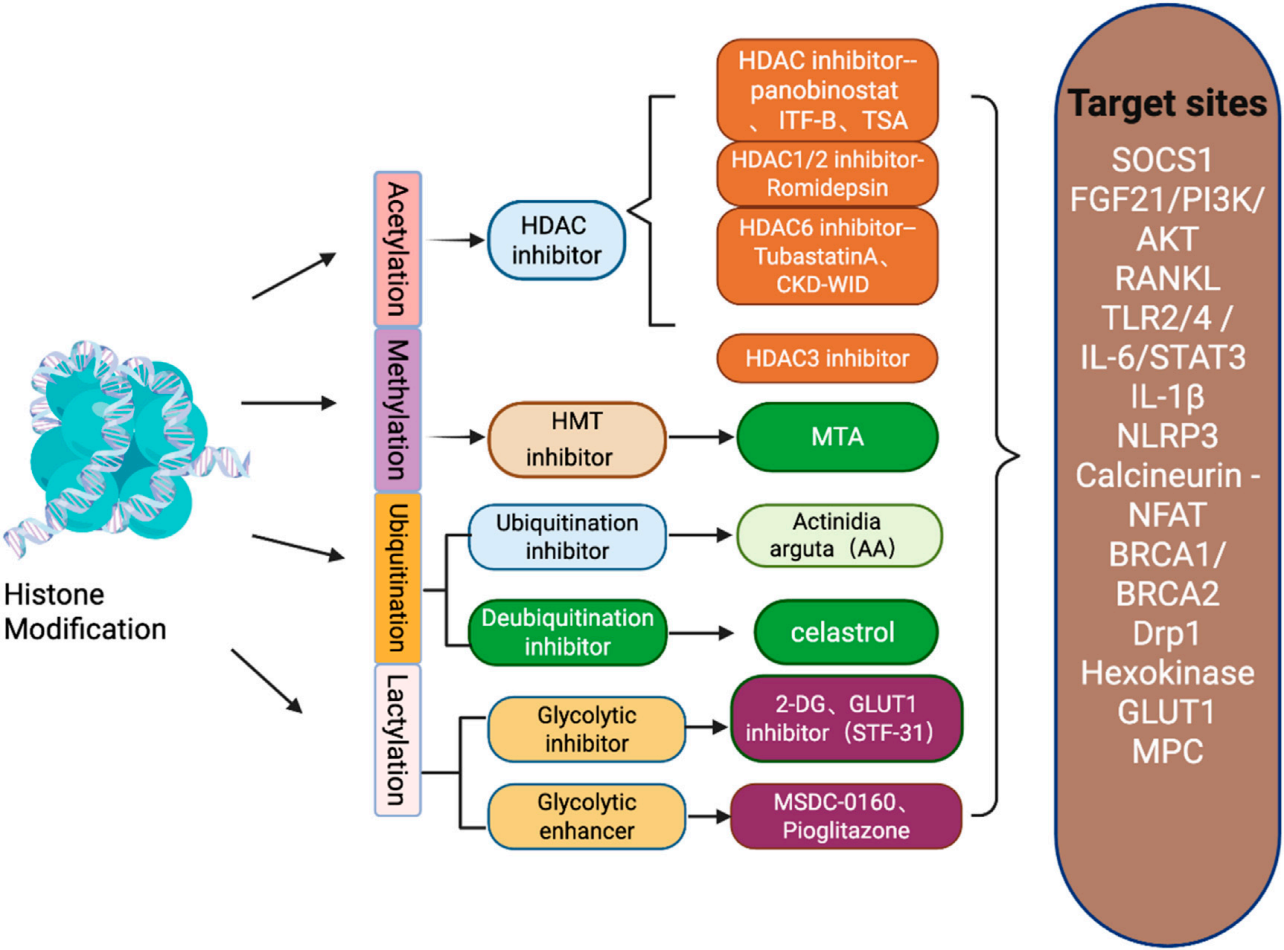
Schematic diagram of histone modification types in gout and inhibitors of various types of modifications and their target sites.

Histone epigenetic modifications are chemical modifications of amino acid residues on histones that regulate gene expression without altering the DNA sequence (Huang et al., 2023). The N-terminal amino acid residues of histones undergo at least 12 types of specific modifications, which influence the binding of nucleosomes to DNA, the three-dimensional structure of chromosomes, and gene expression (Geng et al., 2021). Among these, histone acetylation and histone lysine methylation are the two most typical histone modifications (Figure 2; He et al., 2018). In contrast to genetic factors, epigenetic factors (including histone modifications) exhibit dynamic and reversible characteristics (Shi and Qi, 2023).

Histone methylation is mediated by histone methyltransferases (HMTs), including lysine methyltransferases (KMT) and arginine methyltransferases (PRMT), as well as histone demethylation by histone demethylases (HDM) (Alaskhar Alhamwe et al., 2018). Histone methylation typically occurs through the addition of methyl groups to the lysine (K) residues of histones H3 and H4, making it one of the most important post-translational modifications (Greer and Shi, 2012). Studies on the pro-inflammatory effects of uric acid have focused on the role of monosodium urate (MSU) crystals. However, little is known about whether uric acid itself can directly induce pro-inflammatory effects; our research indicates that high concentrations of uric acid (up to 50 mg/dL) influence inflammatory responses by promoting the production of IL-1β in PBMCs. The mechanism underlying IL-1β amplification involves the downregulation of IL-1Ra, and this effect can be mediated through epigenetic mechanisms such as histone methylation (Crişan et al., 2016), suggesting that histone methylation changes induced by high uric acid concentrations are a key factor in gout attacks. The broad-spectrum protein methyltransferase inhibitor methylthioadenosine (5′-S-methyl-5′-thioadenosine, MTA) has previously been shown to inhibit urate-induced cytokine production *in vitro* (Crişan et al., 2016). This suggests that histone methylation-related processes may play a role in gout.

Histone acetylation modification is an important type of histone modification, indicating increased gene transcription activity and epigenetic marks associated with dynamic chromatin. Histone acetyltransferases (HATs) catalyze the transfer of acetyl groups from acetyl-CoA to the amino acid groups of target lysine residues on histone tails, resulting in the removal of positive charges from histones and weakening the interaction between histones and DNA (which carries negatively charged phosphate groups); HDAC acts as a repressor of gene expression by removing acetyl groups from lysine residues on histone tails (Alaskhar Alhamwe et al., 2018). Histone acetylation can be used to compare gene transcriptional activity, especially H3 acetylation (H3ac) (He et al., 2018). In the context of gout, it has been observed that stimulating macrophages with MSU crystals increases glycolysis, thereby increasing the production of acetyl-CoA, an important product involved in histone acetylation and deacetylation (Cobo et al., 2022). Recent studies have shown that acetyl-CoA plays diverse and important roles beyond intermediate metabolism. In addition to directly regulating metabolic pathways through protein acetylation, CoA also modulates the epigenome through histone acetylation (Theodoulou et al., 2014).

HDACs are believed to play an important role in the activation or silencing of pro-inflammatory genes, and their inhibitors are commonly used to study the pathogenesis of gout.

Notably, it has been demonstrated that treatment of PBMCs with histone deacetylase inhibitors (HDAC inhibitors panobinostat and the potent HDAC inhibitor ITF-B) can suppress inflammatory responses following stimulation with C.16.0 (palmitic acid) and MSU (Cleophas et al., 2016). This finding highlights the potential of using histone modification-related inhibitors as adjunctive therapy for gout. Further studies indicate that, compared to other specific Class I HDAC inhibitors, the HDAC1/2 inhibitor romidepsin is most effective in reducing C16.0+MSU-induced IL-1β production, with its mechanism involving romidepsin upregulating SOCS1 transcription, which has been shown to directly target inflammatory signaling molecules for proteasomal degradation. Therefore, dual HDAC1/2 inhibition may represent an efficient new therapeutic option for acute gout (Cleophas et al., 2019). Uric acid (UA) accumulation triggers endothelial dysfunction, oxidative stress, and inflammation. Histone deacetylases (HDACs) play a crucial role in regulating the pathological processes of various diseases. We administered the broad-spectrum HDAC inhibitor tris(2,2-dimethylamino) propionic acid (TSA) or the selective HDAC6 inhibitor tubastatin A (TubA) to HUVECs or mice, and found that both TSA and TubA reduced inflammation and tissue damage while increasing FGF21 expression and AKT, eNOS, and FoxO3a in the aorta and kidneys of hyperuricemic mice, indicating that HDACs, particularly HDAC6 inhibitors, alleviate UA-induced VECI by upregulating FGF21 expression and subsequently activating the PI3K/AKT pathway. This suggests that HDAC6 may serve as a novel therapeutic target for treating UA-induced endothelial dysfunction (Wang et al., 2023). Additionally, histone deacetylases (HDACs) play a key role in regulating osteoclast differentiation and formation. We investigated the effects of the HDAC6 inhibitor CKD-WID on RANKL-mediated osteoclast formation activation receptor activation in RAW 264.7 mouse macrophages. The results showed that the HDAC6 inhibitor CKD-WID inhibits MSU-induced osteoclast formation by blocking the calcineurin-NFAT pathway in RAW 264.7 cells. This suggests that HDAC6 is a therapeutic target for uric acid-mediated osteoclast generation (Kim et al., 2023). Histone deacetylase 3 (HDAC3) is an essential chromatin-modifying protein in the histone deacetylase superfamily, exerting its transcriptional inhibitory effects through enzymatic histone modifications to maintain normal physiological functions, growth, and development in the body (He et al., 2025). Studies have shown that the absence of HDAC3 in macrophages alleviates MSU crystal-induced gout inflammation by inhibiting the TLR2/4-driven IL-6/STAT3 signaling pathway, suggesting that HDAC3 may serve as a potential therapeutic target for gout (Yang et al., 2024).

It is worth noting that other types of histone modifications in gout remain to be explored, such as lactylation, phosphorylation, and ubiquitination.

Histone lactylation has recently been proposed as a potential mechanism regulating macrophage responses to MSU(de Lima et al., 2023), and changes in intracellular lactate production were observed in MSU-stimulated macrophages through metabolic reprogramming to enhance glycolysis *in vitro* (Renaudin et al., 2020). In another study, it was demonstrated that inhibition of mitochondrial pyruvate carrier (MPC)-induced glycolytic reprogramming in macrophages shifts mitochondrial ATP-related oxygen consumption toward cytoplasmic lactate production, thereby enhancing NLRP3 inflammasome activation by sodium urate (MSU) crystals, suggesting that lactate may be involved in MSU crystal-induced gout attacks (Chen et al., 2023).

Lactic acid drives epigenetic regulation through histone lactylation, activating transcription and cellular plasticity. It acts as a metabolic sensor, translating changes into gene expression (Zheng et al., 2025). Histone lactylation (particularly H3K18la) and lactate co-regulate pathways involving inflammatory mediators such as chemokine signaling and transient receptor potential channels. Reduced H3K18la alleviates symptoms in the CIA model and mitigates the pathogenic effects of TNF-α on FLS, whereas increased H3K18la exacerbates TNF-α-induced pathology (Zheng et al., 2025). Additional studies indicate a positive feedback loop exists between the H3K18la target gene TTK/BUB1B and glycolysis/lactate production. Knockdown of TTK and BUB1B reduces histone lactate synthase (P300) expression, while TTK knockdown inhibits LDHA phosphorylation at Y239. This leads to decreased lactate production and histone lactate levels, thereby affecting the expression of glycolysis-related genes (such as TTK itself) (Li et al., 2024). Therefore, targeting the regulation of glycolysis to alter lactate levels and acidification may serve as an effective therapeutic approach for treating gout attacks.

Felix Renaudin et al. observed that MSU and CPP crystals mediate IL-1β-dependent inflammation during gout and pseudogout attacks by inducing macrophage activation, respectively. The mechanism involves metabolic rewiring toward aerobic glycolysis induced by MSU and CPP crystals, leading to increased lactate. This can be explained by increased GLUT1 plasma membrane expression and glucose uptake on macrophages, and neutrophils isolated from human synovial fluid during gout attacks more frequently express GLUT1 on their plasma membranes than those isolated from blood. Glucose deprivation and treatment with 2-deoxyglucose (2-DG) or the GLUT1 inhibitor (STF-31) both suppressed crystal-induced NLRP3 activation and IL-1β production, as well as microcrystal-induced inflammation *in vivo* (Renaudin et al., 2020). Additional studies have found that inactivating the mitochondrial pyruvate carrier (MPC) via genetic depletion or pharmacological inhibitors such as MSDC-0160 or pioglitazone increases NLRP3 inflammasome activation and IL-1β secretion in macrophages. MPC inhibition exacerbates MSU-induced peritonitis in diabetic mice and increases gout risk in diabetic patients. This mechanism correlates with glycolytic reprogramming and increased lactate production induced by MPC inhibition, indicating that MPC-regulated glycolysis modulates NLRP3 inflammasome activation and gout progression (Chen et al., 2023).

*In vitro* studies have shown that Actinidia arguta (AA) regulates NLRP3 ubiquitination and oligomerization of the CARD (ASC) oligomerization, leading to inhibition of NLRP3 inflammasome-mediated interleukin (IL)-1β secretion. AA provides scientific evidence supporting its traditional claims for treating inflammation and inflammation-mediated metabolic disorders (including gout) (Heo et al., 2018). Another study showed that celastrol can block NLRP3 K63 deubiquitination, which may involve the interaction of celastrol with the BRCA1/BRCA2 complex subunit 3 (BRCC3), thereby preventing the formation of the NLRP3, ASC, and pro-caspase-1 complex, blocking the production of mature IL-1β, and alleviating MSU-induced gouty arthritis (Yan et al., 2021). Studies have shown that the deubiquitinating enzyme USP16 induces gouty arthritis through Drp1-dependent mitochondrial fission and NLRP3 inflammasome activation, with the mechanism being that USP16 mediates Drp1 deubiquitination and stabilization through direct interaction with Drp1 (Wang and Qiu, 2023).

Although histone phosphorylation modifications have not been explored in the context of gout, they may play a role in the inflammatory process alongside other previously reported modifications. Current research on histone modifications in gout has primarily focused on histone acetylation and histone deacetylase inhibitors. This study provides a new scientific perspective for the treatment of gout. In the future, we need to intensify our exploration of other histone modifications in gout, aiming to uncover the mysteries surrounding histone modifications and their mechanisms in gout as soon as possible, and apply these findings to clinical practice to alleviate patient suffering.

### 2.3 Non-coding RNA regulation in gout inflammation

Most of the human genome (76%–97%) encodes RNA that is not translated into proteins, known as non-coding RNA (ncRNA). Since their discovery, the biological importance of ncRNAs has become increasingly apparent, shifting the view of RNA as a simple intermediate in protein synthesis to that of a functional molecule playing a crucial role in gene expression and genomic organization regulation (Nemeth et al., 2024). Recent studies have shown that ncRNA participates in epigenetic regulation by regulating DNA methylation levels or altering histone modifications, thereby influencing the onset and progression of gout (Table 1).

**TABLE 1:** Representative NcRNAs associated with gout pathogenesis and progression.

Non-coding RNA	Expression	Target gene(s)	Biological effects	References
miR-34a	Upregulated	SLC22A12, URAT1	Anti-inflammatory, Lower uric acid	Sun W. F. et al. (2015)
miR-143-3p	Upregulated	GLUT9	Anti-inflammatory, Lower uric acid	Zhou et al. (2019)
miR-223	Upregulated	NLRP3	Anti-inflammatory	Bauernfeind et al. (2012)
miR-223-3p	Upregulated	NLRP3	Anti-inflammatory	Wang et al. (2021)
miR-22-3p	Upregulated	NLRP3	Anti-inflammatory	Wang et al. (2021)
miR-20b	Upregulated	NLRP3	Anti-inflammatory	Rao et al. (2015), Zhang et al. (2019)
miR-488, miR-920	Upregulated	IL-8, TNF-α	Anti-inflammatory	Zhou et al. (2017)
MiR-155	Upregulated	SHIP-1	Promote inflammation	Jin et al. (2014)
miR550a-5p miR550a-3-5p	Upregulated	PSME1, FERMT3, GRK2, OS9	Promote inflammation	Xu et al. (2024)
LncRNA HOTTIP	Downregulation	miR-101-3p/BRD4	Anti-inflammatory	Shao et al. (2023)
lncRNA SNHG14	Downregulation	miR-223-3p	Anti-inflammatory	Yang et al. (2023)
lncRNA H19	Upregulated	APN/PI3K/AKT	Improve lipid metabolism disorders and inflammatory responses in GA	Zhang Q. et al. (2023)
circHIPK3	Upregulated	miR-192, miR-561	Promote inflammation	Lian et al. (2021)
circRNA 104633	Upregulated	JAK2/STAT3	Promote inflammation, Promote a hypercoagulable state	Zhang et al. (2025)
hsa_circRNA_103657 hsa_circRNA_000241	Upregulated	PI3K-Akt	Promote inflammation	Dai et al. (2021)
circRNA novel_circ_0030384	Upregulated	PSME1, FERMT3	Promote inflammation	Xu et al. (2024)
hsa_circRNA_102911	Upregulated	DDX3X\NLRP3\NLRP9	Promote inflammation	Niu et al. (2023)

MiRNAs are non-coding RNA molecules composed of approximately 20 nucleotides (Gebert and MacRae, 2019), which can subtly and complexly regulate gene expression by binding to the 3′-untranslated region of target genes. MiRNAs act as post-translational inhibitors of genes, leading to their degradation or translational inhibition (Yang et al., 2022),interfering with the control of biological processes such as cell proliferation, apoptosis, differentiation, or organogenesis (Tétreault and De Guire, 2013). In recent years, an increasing number of miRNAs have been found to be involved in regulating inflammatory responses in arthritis (such as gouty arthritis, rheumatoid arthritis, and osteoarthritis) (Gao et al., 2018; Xu et al., 2019). MiRNAs can interfere with the abnormal expression of mRNA in inflammatory pathologies, influencing the development of various diseases, including gout (Xu et al., 2020; Huang et al., 2022; Xie et al., 2022). Furthermore, miRNAs can influence serum uric acid and the pathophysiology of gout through different mechanisms, and they can also regulate the expression of uric acid transporter genes. For example, miR-34a targets the mRNA of the SLC22A12 gene and inhibits the expression of URAT1 in a hyperuricemic animal model (Sun R. et al., 2015). MiR-143-3p can directly target the 3′UTR of GLUT9 in renal tubular epithelial cells to reduce uric acid reabsorption and inflammatory responses (Zhou et al., 2019). Additionally, Sun, W. et al. reported that Xie-Zhuo-Chu-Bi-Fang can upregulate miR-34a and downregulate URAT1 to treat hyperuricemia (Sun W. F. et al., 2015). Furthermore, miRNAs can also influence the expression of essential enzymes, such as xanthine oxidase, which can be regulated by miR-448 (Knake et al., 2014). Another mechanism of miRNA interference is to regulate the expression of genes involved in the immune response to gout. For example, miR-223 can inhibit NLRP3 expression and reduce inflammasome activity (Bauernfeind et al., 2012). Furthermore, Wang, X et al. reported that miR-223-3p and miR-22-3p can reduce the inflammatory effects of gout monocytes and mouse models by interacting with the 3′ untranslated region fragment of NLRP3 mRNA (Wang et al., 2021).

In monocytes/macrophages from acute inflammatory lung injury and tuberculosis, MiR-20b is abnormally expressed, indicating its close association with disease inflammation (Rao et al., 2015; Zhang et al., 2019). Whether miR-20b participates and plays a role in the regulation of Nlrp3 expression and inflammatory responses in MSU-induced macrophages has not yet been confirmed. Ya-Fei Liu et al. reported that in synovial mononuclear cells (SFMCs) collected from gouty arthritis patients and MSU-stimulated THP-1 cells, miR-20b was downregulated, while lncRNA HOTAIR and Nlrp3 were upregulated. Treatment with a miR-20b inhibitor significantly increased NLRP3 protein levels and IL-1β and TNF-α secretion in MSU-stimulated THP-1 cells, indicating that miR-20b negatively regulates NLRP3 in an *in vitro* gouty arthritis model. *In vitro* experiments showed that lncRNA HOTAIR expression is regulated by DNA methylation, and HOTAIR knockout inhibits NLRP3 expression and inflammatory cytokine secretion through miR-20b regulation. *In vivo* experiments showed that HOTAIR knockout alleviated ankle swelling in a gouty arthritis mouse model. This study is expected to enrich the existing literature and provide guidance for the treatment of gouty arthritis (Liu et al., 2021). Recent studies have shown that interleukin (IL)-1β is a key inflammatory mediator in acute gouty arthritis (GA), and its levels are regulated by microRNA (miRNA). Weidong Zhou et al. found that upregulation of miR-488 and miR-920 can inhibit IL-1β protein expression in MSU-induced THP-1 cells, but no significant differences in IL-1β mRNA levels were observed; Overexpression of miR-488 and miR-920 significantly inhibited the gene and protein expression of IL-8 and TNF-α in MSU-induced THP-1 cells. These findings suggest that miR-488 and miR-920 could serve as potential therapeutic targets for GA treatment (Zhou et al., 2017).

In summary, these data highlight the ability of miRNAs to act on different targets in gout to achieve urate-lowering and anti-inflammatory effects. Increasing evidence suggests that miRNAs may be potential therapeutic targets for gout. Therefore, research in this field is an important tool for designing new therapies.

Long non-coding RNAs (lncRNAs) are transcripts longer than 200 nucleotides that clearly do not encode proteins. They participate in various functions, and their overexpression, deficiency, or mutation can lead to certain diseases (Bridges et al., 2021). Unlike microRNAs, which primarily rely on RNA sequence complementary pairing to inhibit target genes, lncRNAs operate in a significantly more complex manner. LncRNAs play unique roles in multiple gene expression regulatory mechanisms, including epigenetic regulation, transcriptional regulation, and post-transcriptional regulation (Tan et al., 2020; Fu et al., 2021; Statello et al., 2021). Current research has revealed the axial regulatory mechanism of highly expressed lncRNAs in GA tissues targeting appropriate miRNAs, which subsequently influence GA target genes. For example, lncRNA HOTAIR participates in inflammatory regulation in GA through the miR-20b/Nlrp3 axis (Liu et al., 2021). Ping Shao et al. found that lncRNA HOTTIP promotes the release of pro-inflammatory cytokines interleukin (IL)-1β, IL-8, and transforming growth factor α from MSU-induced macrophages through the miR-101-3p/BRD4 axis, thereby enhancing the inflammatory response in acute gouty arthritis, offering new insights into the diagnosis and treatment of AGA (Shao et al., 2023). Other lncRNA and miRNA interaction patterns are more unique. Through spatial conformation, the secondary structures formed by lncRNAs can exert sponge-like adsorption effects on miRNAs, altering their actual contact concentrations, as well as the production of inflammatory genes or transcription factors in GA tissues (Jiang et al., 2021). For example, in an AGA cell model, the lncRNA SNHG14 acts as a sponge for miR-223-3p, inducing cellular inflammatory responses by regulating miR-223-3p levels, thereby exacerbating the progression of AGA (Yang et al., 2023). Xianheng Zhang et al. found in their study on the use of Huangqin Qingre Baopi Capsules (HQC) for treating GA that HQC improves lipid metabolism disorders and inflammatory responses in GA by regulating the lncRNA H19/APN/PI3K/AKT pathway. Maintaining lipid metabolic stability may be an effective method for alleviating GA (Zhang X. et al., 2023),this provides theoretical guidance for the use of traditional Chinese medicine formulas to regulate lncRNA in the treatment of GA. To date, several studies investigating lncRNA and miRNAs associated with gout have been published. Feng Chen et al. constructed a ceRNA regulatory network and found that five miRNAs (miR-429, miR-137, miR-139-5p, miR-217, miR-23b-3p) and five lncRNAs (SNHG1, FAM182A, SPAG5-AS1, HNF1A-AS1, UCA1) play important roles in the formation and development of gout. And by regulating key lncRNA-mediated signaling axes, they can modulate inflammatory responses, proliferation, differentiation, and apoptosis of chondrocytes and osteoclasts, thereby controlling the formation and development of gout (Chen et al., 2022). These miRNAs and lncRNAs hold promise as molecular markers for gout activity.

In addition, circular RNAs (circRNAs) are non-coding RNAs (ncRNAs) with a single-stranded covalent closed-loop structure, and their abnormal expression may be involved in the pathogenesis of various human diseases. CircRNAs can act as miRNA sponges, interact with RNA-binding proteins, influence protein translation, regulate protein recruitment, and modulate protein assembly (Zhou et al., 2020). Notably, dysregulated circRNAs can exert pathogenic or protective functions in rheumatic diseases, including ankylosing spondylitis (AS), osteoarthritis (OA), osteoporosis (OP), rheumatoid arthritis (RA), systemic lupus erythematosus (SLE), Crohn’s disease (CD), and gout. They primarily promote or prevent disease development and progression through their miRNA sponge activity (Abbas et al., 2023). Although the functions of circRNAs remain incompletely understood, this characteristic makes them potential inhibitors of miRNAs associated with disease progression (Núñez-Carro et al., 2023). Further evidence suggests that circRNAs may have significant potential as biomarkers or therapeutic targets for gout. Fei Dai et al. investigated the use of human circRNA microarrays to identify primary gout patients. Microarray analysis revealed that 238 circRNAs were upregulated in the gout group, 41 circRNAs were downregulated, and correlations were observed between sUA, lipid, and glucose metabolism and circRNA expression levels. This revealed that hsa_circRNA_000241 and hsa_circRNA_103657 are involved in regulating lipid or glucose metabolism in gout patients, and elevated expression levels are associated with risk in gout patients (Dai et al., 2021). Furthermore, circRNA 104633 expression is closely associated with inflammatory and coagulation markers. Xianheng Zhang et al. demonstrated that circRNA 104633 upregulation promotes inflammation and hypercoagulability in gouty arthritis by activating the JAK2/STAT3 pathway. Their study revealed that Jianpi Qingre Tongluo Prescription prevents inflammation and hypercoagulability in GA by inhibiting circRNA 104633 and the JAK2/STAT3 pathway, thereby supporting the development of therapeutic targets and drugs for GA (Zhang et al., 2025). Additional research has revealed that circular RNA circHIPK3 promotes inflammation in gouty arthritis by sponging miR-192 and miR-561, thereby enhancing the expression of their target genes TLR4 and NLRP3. *In vivo* experiments confirm that circHIPK3 knockdown suppresses gouty arthritis (Lian et al., 2021).

Most ncRNAs are regulatory RNAs, including miRNAs, lncRNAs, and circRNAs(Di Mauro et al., 2018). Functionally, miRNAs suppress mRNA translation, regulating gene expression at the post-transcriptional level, while lncRNAs and circRNAs interact with miRNAs, mRNAs, and even proteins. Furthermore, lncRNAs and circRNAs act as competitive endogenous RNAs (ceRNAs) by communicating with mRNAs and competing for shared miRNAs, thereby exerting regulatory roles in gouty arthritis (Ergun and Oztuzcu, 2015; Xu et al., 2024).

However, there has been limited research on the relationship between ceRNAs and gouty arthritis. Bioinformatics analysis indicates that differentially expressed circRNAs appear to participate in the pathogenesis of gout by interacting with multiple signaling pathways, including the FoxO, apelin, and cGMP-PKG pathways (Dai et al., 2021). Reports indicate that dysregulation of the PI3K-Akt signaling pathway may affect uric acid metabolism or gout inflammation (Paré et al., 2021; Zhang et al., 2021). Fei Dai et al. constructed a circRNA-miRNA-mRNA network based on hsa_circRNA_103657 and hsa_circRNA_000241, and performed bioinformatics analysis on their downstream targets. The findings indirectly suggest that dysregulated hsa_circRNA_103657 and hsa_circRNA_000241 may participate in gout pathogenesis by influencing the PI3K-Akt signaling pathway. This provides a theoretical basis for further exploring the potential pathogenic mechanisms of circRNA-miRNA-mRNA regulatory networks in gout (Dai et al., 2021). Yanqiu Xu et al. constructed a co-regulatory ceRNA network of circRNAs and lncRNAs in gouty arthritis, finding that upstream genes in the co-regulatory network (circRNA novel_circ_0030384 and lncRNAs AAMP, TRIM16, PKN1, XLOC_184579, and XLOC_189826) upregulate miR550a-5p and miR550a-3-5p and downregulate PSME1 and FERMT3 expression, and the downregulation of PSME1 and FERMT3 exacerbates gouty arthritis *in vitro*. These genes mediate inflammation in gouty arthritis through chemokine signaling pathways to regulate neutrophil function (Xu et al., 2024). Furthermore, reduced lncRNA expression reversed the inhibitory effect on downstream miRNAs. Decreased expression of novel_circ_0030384 diminished binding with downstream miRNAs and enhanced the suppression of downstream mRNAs by these miRNAs (miR-550a-3p and miR-550a-5p) (Xu et al., 2024). Shaowei Niu et al. analyzed the expression profiles of pyroptosis-related genes (PRG) in peripheral blood mononuclear cells (PBMC) from gout patients using microarray analysis and constructed a ceRNA network to explore the molecular mechanisms of RNA-mediated pyroptosis regulation, identifying hsa_circRNA_102911-has-miR-129-5p-DDX3X\ NLRP3\NLRP9 may be a key regulatory pathway for pyroptosis-mediated regulation of gout inflammation, and hsa_circRNA_102911 may serve as a potential biomarker for diagnosing primary gout (Niu et al., 2023). These studies provide insights into the potential use of circular RNA for the treatment of gout.

## 3 The role of epigenetic remodeling-mediated trained immunity in gout

All organisms frequently encounter various pathogens, ranging from viruses to bacteria, fungi, and worms. The core of the host’s defense against these pathogens lies in the role of immune memory, a long-term property that enables antigen-specific cells of the immune system to recognize and recall information about defending against previously encountered microorganisms. This phenomenon ultimately leads to enhanced and stronger immune responses when subsequently infected with the same pathogen, providing the organism with enhanced survival. Until recently, the consensus was that only T and B cells of the adaptive immune system could generate memory responses, as they are capable of recognizing both specific and diverse antigens (Vuscan et al., 2024). Immune responses in vertebrates are typically divided into innate and adaptive, with only the latter capable of establishing immune memory. However, despite the absence of an adaptive immune response, plants and invertebrates can prevent reinfection by pathogens, and invertebrates even exhibit transplant rejection responses (Netea et al., 2011). In recent years, this dogma has been challenged by the discovery that cells from the innate immune system can also acquire memory-like capabilities following pathogen attack. Bone marrow cells from the innate immune system exhibit increased reactivity when subsequently stimulated with the same or different stimuli (Netea et al., 2020), a phenomenon termed innate immune memory or “trained immunity”. Trained immunity enhances responses to infection and vaccination, promotes stronger innate immune responses, and enhances protection against various microbial stimuli. Additionally, trained immunity may contribute to the pathophysiology of cardiovascular, autoinflammatory, and neurodegenerative diseases (Domínguez-Andrés et al., 2023). Trained immunity, mediated by extensive metabolic rewiring and epigenetic modifications, has significant implications for human disease (Netea and Joosten, 2024). Trained immunity is regulated by epigenetic reprogramming, including changes in histone chemical modifications and chromatin accessibility, which lead to sustained alterations in the transcription of specific genes (Post et al., 2023). Although trained immunity is beneficial against infection, inappropriate induction by endogenous stimuli can lead to abnormal inflammation. For example, in systemic lupus erythematosus and systemic sclerosis, trained immunity may contribute to inflammatory activity, thereby promoting disease progression (Ochando et al., 2023).

We applied this concept to gout, proposing that recurrent gout attacks may be partially attributable to the training effect of prior exposure to high uric acid/uric acid salts on monocyte-macrophages. Evidence supporting this view includes: high concentrations of soluble urate can drive epigenetic reprogramming of myeloid cells, and both *in vitro* and *in vivo* models indicate that cells induced by soluble urate exhibit persistently elevated levels of inflammatory cytokines, increased responsiveness to secondary stimuli, and exacerbated inflammatory symptoms (Crişan et al., 2016), a phenomenon consistent with characteristics of classical trained immunity. Additionally, urate alters the epigenetic landscape of selected human monocytes or whole blood from patients with hyperuricemia. Histone modifications and DNA methylation exhibit urate-exposure-dependent differences, with many inflammation-related genes maintained at a higher state of alertness (Tanaka et al., 2017; Badii et al., 2021).

In population studies, serum from asymptomatic young patients with primary hyperuricemia exhibited molecular features similar to chronic inflammation. Elevated serum uric acid (UA) levels led to enhanced oxidative stress and inflammatory responses, with significant increases in serum malondialdehyde (MDA), IL-6, and TNF-α as UA levels rose, and a significant decrease in superoxide dismutase (SOD) activity (Zhou et al., 2018). Elevated serum urate levels may act as an inducer of subclinical inflammation in AHU subjects, directly inducing the transcription of pro-inflammatory cytokine genes (Luis-Rodríguez et al., 2021). Low-grade inflammation caused by urate crystals in joints may trigger subclinical systemic reactions or may represent systemic spread of local joint inflammation (Inaba et al., 2013). UA is an inducer of monocytes, and multiple studies have shown that UA alters the epigenetic landscape of peripheral blood mononuclear cells (PBMCs) (Li et al., 2023). UA-induced differential changes are reflected in histone modifications and DNA methylation, and these differential changes can lead to persistent tissue inflammation even after MSU crystal dissolution. Additionally, these differential changes demonstrate the potential for UA to alter the epigenetic profile of immune cells. Therefore, the epigenetic changes observed in monocytes could serve as new therapeutic targets for gout treatment (Badii et al., 2021), these findings suggest that hyperuricemia may act as a training factor, conferring memory characteristics to the innate immune system, thereby influencing the frequency and severity of gout attacks.

However, the key question we are now closely monitoring is how long urate crystal-induced trained immunity persists in the human body, whether this innate immune memory requires sustained exposure to a high urate environment, or whether it persists long-term following a single acute episode. M Badii et al. conducted experimental studies using low-concentration uric acid (10 mg/dL) and high-dose uric acid stimulation (50 mg/dL) to induce monocytes, demonstrating that after 24 h, the activation of AKT-PRAS40 and inhibition of autophagy led to changes in cytokine levels produced by cells, with increased IL-1β production and reduced IL-1Ra concentrations. Furthermore, even after extending the rest period between induction and re-stimulation, modified cytokine levels persisted in primary cells exposed to urate. IL-1Ra remained downregulated after a 48-h rest period (Badii et al., 2021). Other reports indicate that innate immune memory induced by β-glucan, BCG, or oxidized LDL particles is best observed when the training interval is 24 h and the resting interval is 6 days, compared to resting for 1 day or 3 days, which may be related to immune metabolic changes (glycolysis induction associated with innate immune memory) (Bekkering et al., 2017). Trained immunity not only drives increased pro-inflammatory responses in monocytes but may also involve enhanced cellular reactivity, such as increased production of pro-inflammatory and anti-inflammatory cytokines, ROS production (in the case of BCG and oxLDL), and amplified metabolic activation (Bekkering et al., 2016). This phenomenon aligns with the characteristics of classical trained immunity.

M Badii et al. demonstrated that pre-treating freshly isolated human peripheral blood monocytes or enriched monocytes with dissolved urate crystals and stimulating them with LPS, with or without monosodium urate (MSU) crystals, resulted in differential expression of histone modifications (H3K4me3 or H3K27ac) and DNA methylation in response to high urate exposure. Uric acid exposure can regulate AMPK and interferon signaling pathways in patients with hyperuricemia through DNA methylation. The broad-spectrum protein methylation inhibitor MTA was shown to reverse uric acid effects *in vitro*, suggesting that epigenetic changes may play a role in mediating the sustained effects of uric acid exposure on innate immune cells. Epigenetic changes in myeloid cells may serve as therapeutic targets for gout (Badii et al., 2021). The above research indicates that urate pretreatment of monocytes can have a sustained impact on innate immune cells through epigenetic reprogramming, including histone modifications and DNA methylation alterations. This epigenetic change can persist even after the dissolution of MSU crystals, leading to the persistent existence of tissue inflammation. It is worth noting that *in vitro* experiments have shown that the protein methylation inhibitor MTA can effectively reverse the effect induced by urate, further confirming that the enhanced cellular inflammatory reactivity triggered by urate treatment belongs to the classic trained immunity rather than a transient inflammatory pre-excitation phenomenon. This discovery provides crucial evidence for understanding the molecular mechanism of gout recurrence.

Notably, Agrawal et al. identified uncertain potential clonal hematopoiesis (CHIP) as a risk factor for gout, particularly mutations in TET2 and DNMT3A, which encode epigenetic regulatory factors (Merriman and Joosten, 2022). TET2 (the primary CHIP gene) and other CHIP genes (e.g., DNMT3A, EZF2) act as epigenetic mediators, increasing the likelihood that molecular mechanisms associated with gout involve training phenomena, where external stimuli (in this case, soluble urate) reprogram the epigenome to train the innate immune system to exhibit a heightened response to MSU crystals (Joosten et al., 2020). This hypothesis can be tested by knocking out the functions of TET2 and other epigenetic CHIP genes, followed by assessing the impact on training phenotypes in an *in vitro* model exposed to soluble urate, including examining effects on the epigenome (e.g., DNA methylation and chromosomal structure) (Badii et al., 2021). In MSU crystal-induced Tet2 knockout mouse models and macrophage studies, exaggerated IL-1β secretion, elevated IL-1β levels, and paw edema were observed, which were improved by genetic and pharmacological inhibition of the Nlrp3 inflammasome. The study revealed that the TET2 mutant CHIP is associated with increased gout risk in humans and identified CHIP as an amplifier of NLRP3-dependent inflammatory responses to MSU crystals in gout patients (Agrawal et al., 2022).

**FIGURE 3:**
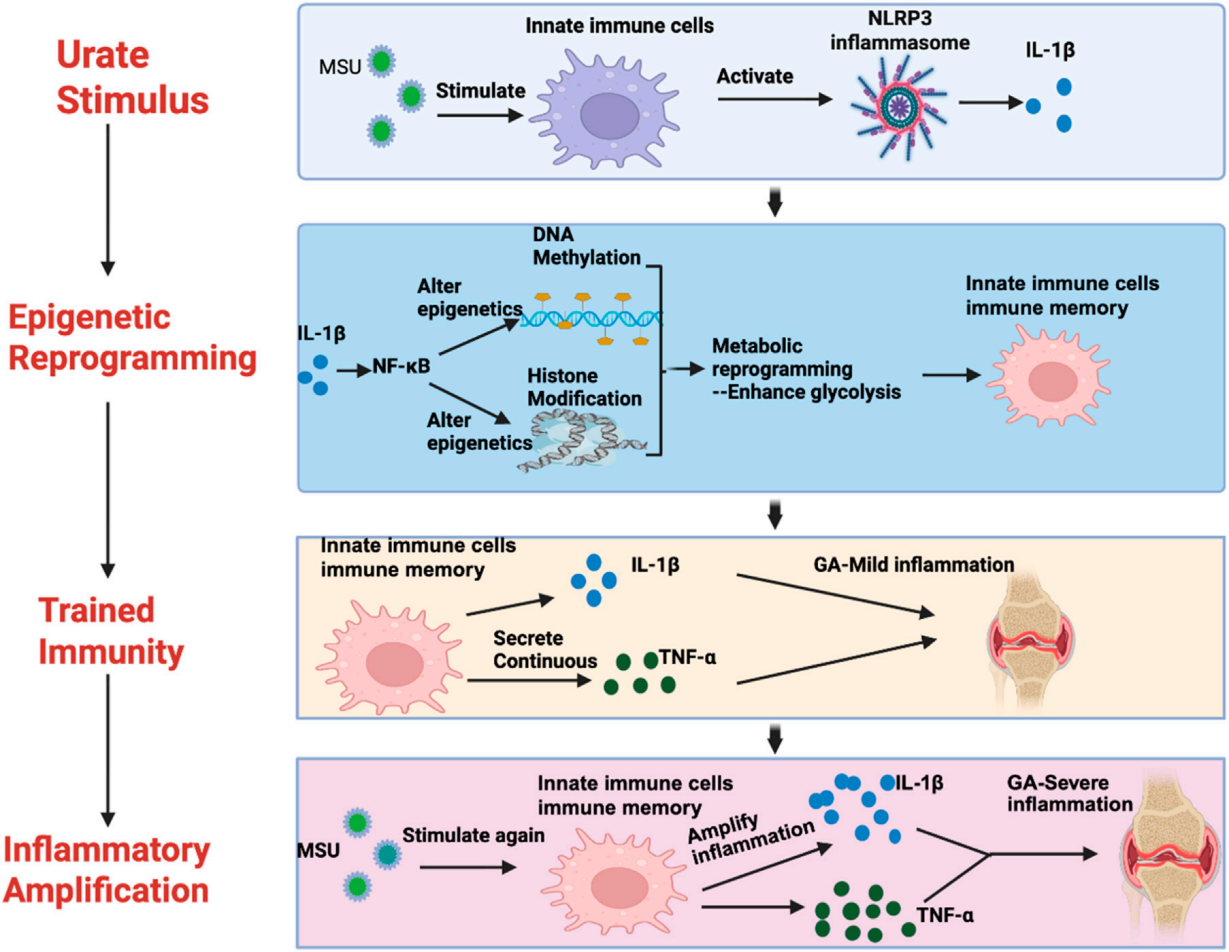
A diagram of the mechanism of trained immunity mediated by epigenetic remodeling in gout.

In summary, we have outlined the fundamental mechanisms of epigenetically mediated trained immunity in gout (Figure 3). Epigenetically mediated trained immunity represents a novel direction in gout research. Moving forward, we must further explore the specific epigenetic pathways and functional targets mediating trained immunity in gout. This will enable us to reduce gout recurrence by intervening in both the epigenetic and trained immunity processes.

## 4 Epigenetic intervention strategies in gout

Based on the above understanding of the mechanisms involved, we explore potential epigenetic intervention strategies for regulating inflammation and immune memory in gout. First, repurposing epigenetic drugs: Some existing epigenetic regulatory drugs (such as HDAC inhibitors, histone methyltransferase inhibitors, etc.) have been used in other diseases (Grandi and Bhutani, 2020) and could be tested for their effects in gout inflammation models.

HDAC inhibitors have been widely used in gout inflammation models. For example, experimental studies have shown that the HDAC1/2 inhibitor romidepsin significantly reduces the production of IL-1β in peripheral blood mononuclear cells (PBMCs) induced by uric acid monosodium crystals (MSU) and palmitic acid (C16.0)-induced IL-1β production in peripheral blood mononuclear cells (PBMCs). The mechanism involves upregulating SOCS1 expression and transcriptionally blocking STAT1 and STAT3 activation, as well as NF-κB-mediated cytokine gene transcription, thereby effectively reducing IL-1β and IL-1β mRNA production (Cleophas et al., 2019). Although romidepsin is a highly effective inhibitor *in vitro*, further research is needed before we can achieve HDAC1/2 inhibition during gout attacks in patients. Additionally, selective HDAC3 inhibitors (such as RGFP966) have been shown to alleviate MSU crystal-induced gout inflammation in mouse models by inhibiting the TLR2/4-driven IL-6/STAT3 signaling pathway, suppressing the production of IL-6 and TNF-α in MSU crystal-treated BMDMs, and shifting the gene expression of BMDMs from pro-inflammatory macrophages (M1) to anti-inflammatory macrophages (M2). Simultaneously, animal model validation showed that HDAC3-deficient mice exhibited significantly reduced inflammation in gout models, providing evidence for the development of selective HDAC3 inhibitors (Yang et al., 2024). In another study, the authors administered UA-induced damaged human umbilical vein endothelial cells (HUVECs) with the broad-spectrum HDAC inhibitor Tricatatin A (TSA) or the selective HDAC6 inhibitor TubastatinA (TubA), or a mouse model of hyperuricemia induced by both potassium oxalate and hypoxanthine. The study demonstrated that both TSA and TubA reduced gout inflammation, tissue damage, and UA-induced vascular endothelial cell damage. The mechanism involved HDAC6 inhibitors upregulating FGF21 expression, which then activated the PI3K/AKT pathway (Wang et al., 2023). In a study exploring the inhibitory effect of butyrate on cytokine production in MSU crystal-induced *ex vivo* cells, the results indicated that the inhibitory effect of butyrate is mediated by inhibition of Class I HDACs, particularly through the inhibition of HDAC1, HDAC2, and/or HDAC3, thereby reducing MSU-induced IL-1β and IL-1β mRNA production in a dose-dependent manner (Cleophas et al., 2016).

Additionally, Kim SK et al. demonstrated in an experimental study using nuclear factor-κB ligand (RANKL) and MSU co-stimulation of RAW 264.7 macrophages to induce osteoclasts that the HDAC6 inhibitor CKD-WID inhibits MSU-induced osteoclast formation by blocking the calcineurin -NFAT pathway in RAW 264.7 cells, thereby inhibiting MSU-induced osteoclast formation. This inhibition reduces the expression of the transcription factor NFATc1 mRNA and nuclear NFATc1 protein, as well as osteoclast-related markers such as c-Fos, TRAP, cathepsin K, and carbonic anhydrase II. This provides theoretical guidance for treating MSU-induced joint bone destruction in gout (Kim et al., 2023). Studies have shown that HDAC6 is considered a therapeutic target for uric acid-mediated osteoclast generation. Therefore, HDAC inhibitors based on epigenetic theory demonstrate significant efficacy in gout inflammation models, offering a new direction for gout treatment.

Secondly, histone methyltransferase inhibitors have also been increasingly applied in various inflammatory models. By using histone methyltransferase inhibitors *in vitro*, urate-induced monocyte inflammation was reversed, indicating that epigenetic changes exist in soluble urate-induced inflammation and can be reversed through intervention (Crişan et al., 2016). The broad-spectrum protein methyltransferase inhibitor methylthioadenosine (5′-S-methyl-5′-thioadenosine, MTA) has previously been shown to inhibit urate-induced cytokine production *in vitro*, reducing the release of IL-1β (Crişan et al., 2016). Simultaneously, MTA inhibited the enhanced joint inflammation and histological effects induced by intra-articular injection of MSU crystals and palmitate (C16:0) in acute gout (Badii et al., 2021). This suggests that histone methyltransferase inhibitors play a prominent role in gout inflammation.

In recent years, research on non-coding RNA has made tremendous progress, miRNAs in non-coding RNA may be involved in the development of inflammatory arthritis, including acute gouty arthritis (Wang et al., 2015). It is conceivable that miRNA mimics or antagonists could be used for therapeutic purposes. For example, overexpression of miR-488 and miR-920 significantly inhibits the gene and protein expression of IL-8 and TNF-α in MSU-induced THP-1 cells, thereby alleviating acute gouty arthritis (Zhou et al., 2017). Related studies have also demonstrated that miR-143-3p can reduce uric acid reabsorption by inhibiting its downstream target gene GLUT9, thereby alleviating inflammation caused by hyperuricemia. Transfection of miR-143-3p mimics into human renal tubular epithelial cells significantly reduced GLUT9 expression. Additionally, inflammatory factors IL-1β and MCP-1 were significantly reduced (Zhou et al., 2019). *In vivo* and *in vitro* experiments demonstrated that miR-223-3p and miR-22-3p inhibit uric acid monosodium-induced gout inflammation by targeting NLRP3 (Wang et al., 2021). Conversely, Jin et al. found that miR-155 expression was elevated in a gout mouse model, and overexpression of miR-155 led to inhibition of SHIP-1 levels and enhanced production of pro-inflammatory cytokines (such as TNF-α and IL-1β),and knocking out miR-155 alleviates inflammation by releasing its inhibition of negative regulatory factors (Jin et al., 2014). However, interestingly, in another study, Qibin Yang et al. found that miR-155 is dispensable in MSU-induced mouse gout inflammation. Deletion of miR-155 may not be an effective treatment for alleviating acute gout inflammation (Yang et al., 2018). Therefore, the role of miR-155 in gout requires further investigation.

Additionally, the co-regulatory ceRNA network mediated by circRNA and lncRNA, which upregulates miR550a-5p and miR550a-3-5p, thereby downregulating the expression of PSME1, FERMT3, GRK2, and OS9, can exacerbate gouty arthritis *in vitro* (Xu et al., 2024). Although there are currently no clinical trials of epigenetic drugs specifically targeting gout, trials of HDAC inhibitors have been conducted in rheumatoid arthritis, showing safety, tolerability, and some anti-inflammatory effects (He et al., 2025). This suggests that epigenetic therapy may have potential applications in inflammatory diseases such as gout, but further optimization of strategies is needed to enhance specificity.

## 5 Conclusion and future perspectives

Summary: Epigenetic regulation of gout and innate immune memory have emerged as hot research topics in recent years. Extensive basic research evidence suggests that high uric acid levels and urate crystals can drive epigenetic reprogramming in immune cells, leading to the sustained “preactivation” of pro-inflammatory genes, thereby establishing an innate immune memory effect in gout patients, subsequently driving the formation of inflammatory memory and gout recurrence. This mechanism helps explain the recurrent acute attacks and chronic low-grade inflammatory state of gout, as well as why some individuals with hyperuricemia are more prone to developing gout. Unlike the traditional perspective that focuses solely on uric acid production and deposition, the epigenetic and immune memory perspective offers new insights into the prevention and treatment of gout. However, the existing evidence system has limitations. Current research overly relies on *in vitro* models of monocytes stimulated by high concentrations of urate crystals or association analyses in hyperuricemic populations, lacking direct *in vivo* experimental data to validate the hypothesis. For instance, whether urate-induced epigenetic reprogramming *in vivo* exhibits persistence and specificity requires establishing causality through controlled animal models. *In vitro* models struggle to replicate complex *in vivo* microenvironments (e.g., intercellular signaling networks, metabolic dynamic regulation), while population studies suffer from confounding variables (e.g., genetic background, comorbidities). Furthermore, the criteria distinguishing trained immunity from inflammatory priming remain unclear—the latter manifests as transient functional enhancement, while the former requires demonstrating persistent epigenetic imprints. This translational gap casts doubt on the hypothesis’s clinical applicability, urgently necessitating direct validation of trained immunity’s molecular mechanisms through controlled *in vivo* experiments. To bridge the translational gap between *in vitro* and *in vivo* studies of the trained immunity hypothesis, priority should be given to designing animal model-based experiments for direct evidence. For instance, the hypothesis could be validated through the following strategies: First, epigenetic tracing—systematically detecting persistent changes in trained immunity-related genes (e.g., H3K4me3 modifications in IL-6 and TNF-α promoter regions) using ChIP-seq in MSU-induced mouse gout models, followed by longitudinal comparisons with acute LPS inflammation models. Second, functional validation integration: Combine multi-omics analyses (e.g., ATAC-seq, RNA-seq) with flow cytometry to establish causal relationships between epigenetic modifications and enhanced immune cell function.

Prospects: Future research should further deepen our understanding of the mechanism: for example, mapping the dynamic changes in the epigenetic profiles of immune cells in gout patients to identify which “training” markers are associated with disease recurrence. Additionally, translating basic findings into clinical applications is needed: exploring epigenetic markers as biomarkers for gout susceptibility and attack prediction, and assessing the feasibility of epigenetic interventions in clinical settings. Of particular note, personalized treatment may benefit from this—customizing different preventive strategies based on patients’ epigenetic and inflammatory memory states (e.g., for patients exhibiting significant trained immune phenotypes, early application of intervention strategies to weaken their inflammatory memory and prevent frequent flare-ups). Currently, epigenetic research in the pathogenesis of GA is still in its infancy, and more studies are needed to further analyze its role in GA progression. Although histone acetylation inhibitors, particularly HDAC1 antagonists, have shown promising anti-inflammatory effects in the laboratory, further experiments are needed to validate their therapeutic effects in clinical settings. Additionally, studies investigating the roles of other histone modifications (such as ubiquitination and phosphorylation) in GA represent potential research directions. Furthermore, several key questions regarding epigenetics in gout remain unresolved. One key issue is the timing of these epigenetic changes, specifically whether they occur in the early stages of disease progression. For example, one hypothesis suggests that changes in the epigenetic landscape occur in the early stages of GA, prior to the onset of symptoms. This would directly impact the timing of treatment, and future studies will need to test whether the epigenome can be reprogrammed in the late stages of the disease. Another common issue in GA drug development is patient heterogeneity, which may be particularly pronounced for epigenetic changes, as the activity of regulators or target factors may vary between patients. In summary, incorporating epigenetic regulation and innate immune memory into the gout research framework not only enriches our understanding of the pathogenesis of gout but also provides a cutting-edge direction for developing novel therapeutic interventions.
